# Effect of GABA-Fortified Oolong Tea on Reducing Stress in a University Student Cohort

**DOI:** 10.3389/fnut.2019.00027

**Published:** 2019-03-26

**Authors:** Tina Hinton, Herbert F. Jelinek, Vincent Viengkhou, Graham A. Johnston, Slade Matthews

**Affiliations:** ^1^Pharmacology, School of Medical Sciences, University of Sydney, Sydney, NSW, Australia; ^2^Clinical Medicine, Macquarie University, Sydney, NSW, Australia; ^3^School of Community Health, Charles Sturt University, Bathurst, NSW, Australia

**Keywords:** oolong tea, GABA, heart rate variability, stress, autonomic nervous system

## Abstract

GABA-containing tea has gained popularity as an accessible intervention to reduce the impact of chronic stress-induced autonomic imbalance and increased risk for cardiovascular disease despite a lack of evidence concerning the γ-aminobutyric acid (GABA) content in a cup of the tea and its effects on physiological and psychological stress as measures of cognitive function. We aimed to measure the effects of GABA-fortified tea consumption on heart rate variability (HRV) and stress in 30 participants using a pre-post cohort study design. Ten minute lead II ECG recordings were analyzed with Kubios software. Frequency domain parameters including total power, high and low frequency power, along with heart rate, were determined. A control group that consumed a non-fortified tea was included in the research. Statistical analysis was by two-way ANOVA for two-group comparison with time as an interaction and a significance level of *p* < 0.05. Oolong tea consumption led to a significant decrease in the immediate stress score and a significant improvement in HRV. We conclude that autonomic imbalance and HRV in people with acute stress is significantly reduced following a cup of GABA fortified oolong tea and highlights the complex interaction between autonomic nervous system function and mood.

## Introduction

Stress is defined as a disruption of the body's homeostasis. Activation of the stress response is crucial for survival by enabling an organism to cope with and adapt to internal and external factors. Stressors may be psychological or physiological, or both. The body's ability to maintain homeostasis in the face of continually changing environmental circumstances and stressors is controlled in part by the autonomic nervous system, and the balance between parasympathetic and sympathetic innervation of viscera, vasculature, the heart, skeletal muscle, and the control of energy metabolism (1). Stress results in sympathetic nervous system (SNS) activation and parasympathetic nervous system (PNS) withdrawal. Input by the sympathetic and parasympathetic branches of the ANS to the heart lead to a continuous change in heart rate that can be expressed as alterations in heart rate variability (HRV). Chronic or acute stress leads to ANS imbalance and decreased HRV (2, 3). Importantly, HRV measures fluctuations in heart rate, which are used as an indicator of autonomic involvement in the control of the heart (4–6). HRV is defined as the statistical variability of the time interval between consecutive heartbeats, that is, between successive *R* waves of an electrocardiograph (ECG) the RR interval (7). Many physiological factors are involved in controlling heart rate, including cardiac sino-atrial node innervation by SNS efferent fibers and the PNS vagus nerve, which decrease and increase the RR interval length, respectively. Each of these factors inherently produces a characteristic and distinct frequency of fluctuation in the RR interval (8, 9), with the overall RR interval series representing their summation. In this way HRV represents a sensitive indicator of adaptability to stress (10), and therefore provides a useful measure of stress response. Cognitive therapy and pharmacological intervention are the mainstream treatment options for anxiety and stress but are often not accessible to all experiencing these conditions (11).

HRV can be analyzed using frequency domain analysis, which analyses the components of the total power in sinus rhythm. Total power indicates the total variability within the heart rate tachogram and can be subdivided into high frequency and low frequency components. High frequency power indicates parasympathetic function, however low frequency power reflects a combination of sympathetic and parasympathetic activity (12).

Tea (*Camellia sinensis*) is reported to be the most widely consumed beverage worldwide (13–15) and has been shown to reduce physiological stress and anxiety, and induce relaxation (14, 16–19). Green tea consumption was shown to improve relaxation in women who enjoyed high social support at work (20), and 6 weeks of black tea consumption led to reduced cortisol levels, and increased subjective relaxation following an acute stress task in healthy non-smoking men (18). Mice exposed to green tea extract perinatally showed reduced anxiety and fear responses on the elevated plus maze (21). Further, decaffeinated tea reduced blood pressure and lowered arousal in mice (17). GABA-fortified green tea was also shown to reduce behavioral indicators of depression and stress in mice in a forced swim test (19). Tea contains numerous constituents including polyphenols such as epigallocatechin gallate (EGCG), amino acids including theanine and GABA, and purine alkaloids like caffeine (13, 14, 22) that may facilitate the effects of tea on stress and mood.

A popular intervention in complementary medicine practices to improve chronic stress involves dietary supplementation with γ-aminobutyric acid (GABA) (23). The effect of GABA on stress reduction is due to both peripheral, acting on the autonomic nervous system ganglia (24), and central processes (25). One such commercially available product is GABA-enriched oolong tea. HRV, as a measure of the stress response, provides an ideal tool with which to investigate the anti-stress or antianxiety effect of GABA fortified tea. HRV is regulated via a feedback loop by higher order nervous system processes including cortical, subcortical and brainstem areas (26). Due to this central and peripheral interaction, chronic psychological stress is able to induce changes in HRV (3). GABA content in tea or other consumables is considered an option to reduce this risk.

This study aimed to investigate the effect of GABA-enriched tea on HRV, and on subjective stress.

## Methods

All experimental procedures were conducted in a quiet room with air-conditioning set at 22° Celcius and ambient light controlled via a dimmer switch, between 10 am and 12 noon to minimize the effects of external stimuli and diurnal HRV fluctuations (27). Upon arrival, participants were randomly allocated into either a GABA-fortified oolong tea group or control group. Both groups habituated to the environment for 10 min, during which time they completed the pre-tea acute stress score as a measure of immediate stress state and Cohen's Perceived Stress Scale (PSS) (28) as a measure of chronic stress status. A 10-min baseline supine ECG was then recorded, as detailed in Experimental measures. The GABA-fortified oolong tea was then consumed over a maximal 5-min period by the GABA-fortified group. After 30 min, a second supine 10 min ECG was recorded, after which the post-tea immediate stress score was completed. The same protocol was followed for the control group who consumed a non-GABA-fortified tea.

### Participants

Thirty healthy volunteers (11 males, 19 females) were recruited from the student cohort at the University of Sydney by advertisements posted around the university. Inclusion criteria were: age 18-30 years, non-smoker, no history of CVD or diabetes, not taking any medications and not pregnant. Group number was determined by a power analysis with Type 2 error set at 0.8, a median effect size and *p* < 0.05. This gave a suggested participant number of 27 (29). Thirty participants were included in the study and the female to male ratio nearly equal (not significant). Participants were instructed to refrain from consuming alcohol or caffeine or engaging in strenuous physical activity, for 3 h prior to the study. This study was carried out in accordance with the recommendations of the NHMRC Human Research Ethics Guidelines and the University of Sydney Human Research Ethics Committee with written informed consent from all subjects. All subjects gave written informed consent in accordance with the Declaration of Helsinki. The protocol was approved by the University of Sydney Human Research Ethics Committee (approval number: 12715).

### Tea Preparation

Oolong tea was sourced commercially from Taiwan and prepared fresh for each participant by adding a standard cup volume (200 mL) of tap water at 90° Celcius to 5 g of tea leaves. The tea was allowed to steep for 10 min before being strained into a cup, left to cool for 10 min and served. This protocol was designed to the manufacturer's recommendations to replicate consumer practices. High performance liquid chromatography showed the GABA content of the GABA-enriched tea to be 2.01 mg/200 mL, while that of the regular oolong tea was 0.25 mg/200 mL.

### Experimental Measures

A pre-test post-test study design was used for this research. A self-assessment, single item of immediate stress level (hereon referred to as the immediate stress score) was conducted before and after tea consumption by asking the participant to rate “how stressed you feel right now, at this exact moment in time” on a scale of 1 (“relaxed”) to 10 (“highly stressed”) after participants were accustomed to the recording room and recording apparatus at a previous visit. This validated single item stress assessment was adapted from Elo et al. (30). Chronic stress levels were assessed using the PSS (28), a 14-point questionnaire validated to quantify the degree to which the participant perceived their life over the past month as stressful. Participants were divided into high or low chronic stress groups based on whether their PSS score was above or below the sample median of 25.5. HRV was the other main outcome measure determined from a 10 min ECG recording using a standard three lead configuration representing Einthoven's triangle (31). Lead I was selected as the monitoring lead unless a well-defined R wave could not be detected, whereby Lead II was used instead. Following removal of ectopic beats, time and frequency domain parameters were determined including the average heart rate, total power, high frequency and low frequency power of the beat-to-beat interval changes over the 20 min recording period.

Upon arrival, participants were provided 10 min to habituate to the environment, during which they completed the Perceived Stress Score and pre-tea acute stress score. A 10 min baseline supine ECG was then conducted. Participants were then provided 5 min to consume their allocated tea, which they were blinded to. After 30 min, a second supine 10 min ECG was conducted, after which the post-tea acute stress score was completed. During all ECGs, participants were instructed to lie with their arms by their sides with eyes open and to refrain from moving or speaking or intentionally altering their respiration. Importantly, reviewing the overall range of RR intervals from participants indicated no outliers, which would have suggested other variables such as respiration contributed to HRV measures.

Data were recorded using a PowerLab 2/20 (ADInstruments; Sydney, Australia) at a sampling rate of 1 kHz and analyzed using Chart version 7 with HRV Module (ADInstruments). A 3 Hz high-pass filter removed wandering baselines while a 45 Hz low-pass filter removed power supply interference. In accordance with recommendations of the Task Force (1996), each ECG was manually inspected for missing and incorrectly classified beats, which were corrected. Remaining ectopic beats were replaced by linear interpolation (32), a method with minimal impact on the underlying HRV structure (14), and the edited RR interval series exported to Kubios HRV version 2.0 (Biosignal Analysis and Medical Imaging Group, Department of Physics, University of Kuopio) for subsequent analyses (33). As the RR interval series intrinsically consists of samples unequally spaced in time, it was interpolated and resampled at 4 Hz to produce the equally sampled data points required by the Fast Fourier Transform (Welch window, width 256 s, 50% overlap) to determine total power (TP), low frequency power (LF), high frequency power (HF), the LF:HF ratio of the power spectral density associated with the RR interval tachogram. Frequency domain analysis was chosen as it has been shown to best represent parasympathetic and sympathovagal balance of the autonomic nervous system modulation of the heart. Table 1 summarizes the various parameters arising from frequency domain analyses of HRV data to provide their physiological meaning with respect to stress.

**Table 1:** Physiological meaning of HRV parameters and their relation to stress.

**HRV parameter**	**Physiological meaning**	**Normal values (34)**
Total power (TP)	Overall variability (ANS functionality and adaptability). Reduced under stress (35)	3466 ± 1018 ms^2^
Low frequency power (LF)	Baroreflex (SNS and PNS activity depending on orthostatic state). Variable effects under stress depending on posture (26)	1170 ± 416 ms^2^
High frequency power (HF)	PNS activity. Reduced under stress (26)	975 ± 203 ms^2^
Ratio of low frequency to high frequency power LF/HF	SNS activity and/or sympathovagal balance (controversial). Increased under stress (36)	1.5 – 2.0

### Statistics

Complete data for all participants were analyzed using SPSS version 22.0.0 (IBM; Chicago, IL, U.S.A.) and are reported as mean ± standard deviation. Normality of the data was determined by the Kolmogorov-Smirnov test. The immediate stress score, TP, LF, HF, and LF:HF were not normally distributed and thus log transformed prior to analysis. To determine the effect of tea consumption on HRV and immediate psychological stress, data were analyzed using a mixed two-way between-subject (type of tea) × within-subject (time: pre, post) ANOVA design. The repeated measure dependent variable was either the immediate stress score or the HRV parameters before and after tea consumption (herein referred to as the factor of time). Threshold for significance was set at *p* = 0.05

## Results

Table 1 shows the baseline characteristics for the 30 participants divided by tea consumed (collapsed across stress level) or by stress level (collapsed across tea consumed).

**Figure 1:**
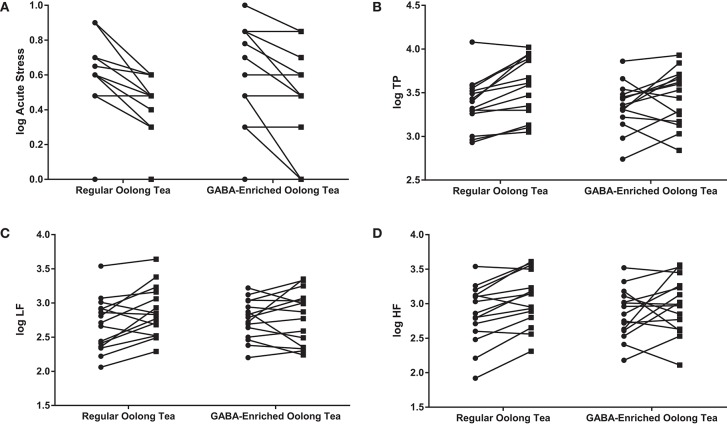
Changes in **(A)** the acute stress score, **(B)** TP, **(C)** LF, and **(D)** HF in the GABA-enriched and regular oolong tea groups. Data are expressed as log values. Each line represents a separate participant. 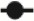 indicates pre-tea value; 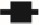 indicates post-tea value.

A significant main effect of time was found for the decrease in immediate stress score (0.547 ± 0.279 to 0.387 ± 0.256; *F* = 34.422, *p* < 0.001) and increase in TP (3.364 ± 0.276 to 3.519 ± 0.331; *F* = 15.221, *p* < 0.001), LF (2.726 ± 0.338 to 2.834 ± 0.374; *F* = 5.564, *p* < 0.05) and HF (2.852 ± 0.385 to 3.022 ± 0.397; *F* = 9.343, *p* < 0.005). Figure 1 shows this effect of time for each participant grouped according to tea consumed.

The immediate stress score was significantly higher (*t* = −4.000, *p* < 0.05) and LF:HF significantly lower (*t* = 2.234, *p* < 0.05) in the high compared to the low stress group. No other comparisons were significant after confounder variables including age were considered. The mean immediate stress score, average RR interval, TP, LF, HF, and LF:HF before and after tea consumption, with subjects divided by tea and stress level, are shown in Table 2.

**Table 2:** Sample characteristics at baseline as divided by tea consumed or chronic stress level.

**Parameter**	**Tea consumed**		**Chronic stress level**	
	**Control (*n* = 15)**	**GABA-enriched oolong (*n* = 15)**	**Low (*n* = 15)**	**High (*n* = 15)**
Gender (% male)	33	40	47	27
Age (years)	21.00 ± 1.414	21.73 ± 1.710	21.27 ± 1.534	21.47 ± 1.685
PSS score	23.47 ± 8.551	28.87 ± 9.094	19.20 ± 5.401	33.13 ± 6.186^*^
Acute stress score	0.537 ± 0.259	0.557 ± 0.306	0.381 ± 0.270	0.712 ± 0.171^*^
Average RR interval (ms)	856.8 ± 120.0	864.1 ± 203.3	829.9 ± 112.5	891.0 ± 202.8
TP	3.379 ± 0.293	3.349 ± 0.268	3.377 ± 0.325	3.350 ± 0.228
LF	2.689 ± 0.387	2.762 ± 0.289	2.811 ± 0.380	2.641 ± 0.276
HF	2.849 ± 0.423	2.854 ± 0.357	2.816 ± 0.471	2.887 ± 0.287
LF/HF	−0.159 ± 0.314	−0.092 ± 0.322	−0.006 ± 0.325	−0.246 ± 0.260^*^
**UNTRANSFORMED VALUES**				
Acute stress score	3.97 ± 0.522	4.40 ± 0.675	2.83 ± 0.401	5.53 ± 0.559
TP (ms^2^)	3026 ± 689	2636 ± 420	3099 ± 698	2563 ± 399
LF (ms^2^)	729 ± 214	699 ± 111	909 ± 215	520 ± 80
HF (ms^2^)	1017 ± 222	973 ± 217	1023 ± 234	967 ± 204
LF/HF	0.880 ± 0.189	0.996 ± 0.160	1.218 ± 0.207	0.658 ± 0.091

* *Significantly different (p < 0.05) than the corresponding value in the low chronic stress group*.

Only the average RR interval showed a significant time x tea x stress level interaction (*F* = 4.977, *p* < 0.05). The regular oolong tea increased the average RR interval by 83.0 and 39.2 ms, and the GABA-enriched oolong tea by 49.2 and 85.6 ms, in the low and high stress groups, respectively (Table 3).

**Table 3:** Psychological stress and heart rate variability parameters before and after ingestion of either GABA-enriched or control tea, separated by low, or high chronic stress level.

**Parameter**		**Regular tea**		**GABA-enriched oolong tea**	
		**Low stress (*n* = 8)**	**High stress (*n* = 7)**	**Low stress (*n* = 7)**	**High stress (*n* = 8)**
Immediate stress score	Pre	0.396 ± 0.253	0.698 ± 0.159	0.365 ± 0.308	0.724 ± 0.192
	Post	0.307 ± 0.218	0.476 ± 0.107	0.197 ± 0.261	0.553 ± 0.272
Average RR (ms)	Pre	849.8 ± 130.7	864.8 ± 116.4	807.2 ± 92.1	914.0 ± 263.3
	Post	932.8 ± 134.3	904.1 ± 83.2	856.4 ± 112.7	999.5 ± 242.6
TP (ms^2^)	Pre	3.397 ± 0.365	3.358 ± 0.210	3.355 ± 0.301	3.343 ± 0.258
	Post	3.537 ± 0.342	3.649 ± 0.355	3.396 ± 0.248	3.494 ± 0.378
LF (ms^2^)	Pre	2.839 ± 0.405	2.518 ± 0.305	2.778 ± 0.379	2.748 ± 0.209
	Post	2.937 ± 0.387	2.795 ± 0.361	2.752 ± 0.376	2.837 ± 0.420
HF (ms^2^)	Pre	2.809 ± 0.535	2.894 ± 0.281	2.825 ± 0.428	2.880 ± 0.311
	Post	2.995 ± 0.464	3.154 ± 0.317	2.819 ± 0.458	3.113 ± 0.320
LF:HF	Pre	0.031 ± 0.215	−0.376 ± 0.271	−0.047 ± 0.453	−0.132 ± 0.201
	Post	−0.059 ± 0.31	−0.359 ± 0.197	−0.067 ± 0.544	−0.276 ± 0.121
**UNTRANSFORMED VALUES**					
Immediate stress score	Pre	2.81 ± 1.252	5.29 ± 1.976	2.86 ± 1.952	5.75 ± 2.435
	Post	2.25 ± 1.035	3.07 ± 0.732	1.86 ± 1.125	4.13 ± 2.100
TP (ms^2^)	Pre	3497 ± 3584	2489 ± 995	2645 ± 1269	2629 ± 1978
	Post	4424 ± 3206	5636 ± 3413	2832 ± 1432	4098 ± 2752
LF (ms^2^)	Pre	1014 ± 1056	403 ± 266	788 ± 538	622 ± 322
	Post	1266 ± 1357	844 ± 760	756 ± 576	988 ± 816
HF (ms^2^)	Pre	1092 ± 1088	932 ± 577	945 ± 720	998 ± 980
	Post	1543 ± 1407	1805 ± 1368	1037 ± 1109	1619 ± 1113
LF:HF	Pre	1.227 ± 0.854	0.484 ± 0.248	1.208 ± 0.802	0.810 ± 0.371
	Post	1.103 ± 0.589	0.476 ± 0.201	1.284 ± 0.883	0.548 ± 0.148

*Light gray shading indicates a significant (p < 0.05) time x tea x stress level interaction*.

## Discussion

This study aimed to measure the effects of GABA-fortified vs. regular oolong tea consumption on HRV and self-reported stress in 30 young participants using a pre-post cohort study design. This is not the first study examining GABA-fortified supplementation and its effect on the stress response. However, it is the largest study to report an effect of GABA-fortified oolong tea and effect on stress as measured by HRV and self-reported stress levels.

A significant increased main effect of oolong tea for HRV and acute stress score (decreased) was found. The significant time x tea x chronic stress level interaction observed for the average RR intervals suggests that in both stress groups, both teas raised the average RR intervals. An increase in RR interval reflects more stable autonomic function through an increase in vagal activity (elevated PNS activity), reflective of a reduction in stress response (8, 35). However, the greater influence of GABA-enriched tea on overall HRV (measured by a change in the RR interval) compared with regular tea for highly chronically stressed respondents suggests an additional benefit of GABA-fortification in tea for this group of individuals. The greater influence of the regular oolong tea in the low, chronic stress group further indicates that the different teas do affect autonomic cardiac control to a different extent (when chronic stress level is also considered), providing a rationale for further studies. Moreover, this finding suggests that other active constituents in oolong tea in addition to GABA have stress-reducing effects.

“The ability for GABA to reduce stress levels depends in part on its capacity to cross the blood brain barrier and to affect neurons in areas such as the amygdala, as well as in the paraventricular nucleus of the hypothalamus and monoamine brainstem nuclei where GABA inhibits sympathetic outflow (15). The finding of a GABA-transporter in the brain suggests that GABA can cross the blood brain barrier and therefore central action of GABA supplementation is likely (37). Nonetheless there are no data on GABA blood-brain-barrier permeability in humans, and whether oral GABA consumption increases its concentration in the brain. However, there is some evidence that GABA oral supplementation reaches the brain in concentrations able to exert biological effects in humans. For example, 100 mg GABA in water increased the alpha:beta EEG wave ratio compared with water alone, suggesting improved relaxation (38) and chronic GABA tea consumption increased sleep efficiency and reduced latency to sleep onset (39). Further understanding of GABA bioavailability following oral consumption is required. GABA may also reduce stress levels by acting on GABA receptors located on sympathetic pre- and post-ganglionic neurons where they inhibit noradrenaline activity (18–21). In addition GABA has been shown to activate extrasynaptic GABA_A_ receptors to generate a long lasting inhibitory state (40). As GABA is extensively distributed within the enteric nervous system, it has been suggested that a bidirectional activity or modulation via the PNS through the vagus nerve could also affect stress responses (41), though this requires further investigation.

Of interest in the current research is that the concentration of GABA in the GABA-fortified tea sample (2.01 mg/200 mL which equates to 40.2 mg/100 g as determined by HPLC) was less than previously reported in similar research but still led to a positive response. The significant increased HRV, measured as total power, indicates elevated overall variability, associated with relaxation, while the rise in the high frequency component of the HRV power spectrum suggests improved parasympathetic tone (12, 26, 35, 36). This stress-reducing effect is reflected in the significant decreased immediate stress score. Thus, the experimental protocol of this study, which involved consumption of GABA-fortified and regular oolong tea, induced HRV changes (suggestive of correction of stress-induced autonomic nervous system imbalance) and reduction of psychological stress.

Delaney and Brodie showed that an acute stressor (mental arithmetic) led to a significant decrease in total power and the high frequency component with a concurrent increase in the LF:HF ratio (42). This reflects parasympathetic withdrawal and greater sympathetic regulation typical of the acute stress response (36, 42–44). Comparatively, chronic stress as measured by the PSS (45), a visual-analog scale (46) or trait anxiety (47–49) was associated with reduced total power and parasympathetic tone. Combined, these findings suggest that under prolonged psychogenic stress, the sympathetic hyperactivity of the acute stress state is attenuated, while parasympathetic withdrawal persists. Thus, it follows that tea consumption under the setting of the present study, increased HRV features that indicate parasympathetic tone, while those proposed to indicate primarily sympathovagal balance (such as LF:HF) remained unaffected.

The HRV effects observed in this study differ from those previously reported for GABA-fortified foods. Okita and colleagues assessed the effects of a single dose of the vegetable kale (containing 31.8 mg GABA), formulated into a tablet compared to a control tablet with no GABA, on chronic stress-induced changes in HRV (50). GABA attenuated the increase in LF and LF:HF reported in the control group without affecting HF. GABA reduced the LF without a detectable effect on peripheral nervous system activity. In support of this finding, oral ingestion of 50–100 mg GABA in water reduced salivary chromogranin A, a protein co-released with noradrenaline in the SNS, indicating a reduction of SNS activity (51). In contrast, both tea options in the present study exerted their effects by parasympathetic augmentation with comparatively smaller sympathetic effects.

In humans the minimum effective oral dose of GABA leading to HRV changes was suggested at 20–30 mg (52). Interestingly in our study the concentration of GABA in the tea was much lower, yet a reduction in perceived immediate stress decreased and HRV parameters improved. This reduction may however have also been due to the experimental condition or other bioactive constituents in the tea (53). Two main bioactive components of tea that may have contributed to the observed stress-reduction are (–)-epigallocatechin gallate (EGCG) and L-theanine. Both are able to cross the blood-brain barrier (54, 55) and are therefore able to influence the central stress processes, which regulate HRV. Both have also shown anxiolytic effects in humans and animals (36, 40–45). On the other hand, teas are known to contain caffeine. Caffeine in tea has also been shown to weakly inhibit GABA_A_ receptors (56). Weak inhibition may have a mildly stimulating effect. It has also been suggested that the stimulant effects of tea may be due, in part, to the caffeine (57). Our results suggest, however, that the observed stress-reducing effects of tea are not contributed to by any potentially stimulating effects of caffeine.

As teas contain a number of active constituents, including catechins, theanine and caffeine, the concentrations of these may differ between GABA-fortified and non-fortified teas owing to the additional processing required to increase GABA content in the GABA-fortified tea (19). It cannot be ignored that differences observed in the effects of the two teas may be due to differences in concentrations in other active constituents in the teas. Further studies to evaluate the concentrations of these active constituents in the teas are warranted, and blood samples for measuring their serum concentrations in participants would also be valuable.

In future studies it will be useful to ascertain the regularity of consumption of teas and other stimulant beverages by participants as a further mediating factor in our findings. Blood pressure would further provide a useful measure of stress, although it is difficult to ascertain blood pressure acutely without influencing the stress state. Glycaemia is another important consideration which may impact results and would be a valuable measure in future studies via a simple finger prick test. While studies of acute GABA or GABA-fortified food and beverage consumption have not documented side effects, the safety and tolerability of GABA consumption and overconsumption should also be considered in future investigations.

In conclusion, our results indicate that even a very small amount of GABA-fortified oolong tea leads to a significant effect on acute stress levels reflected by the improved HRV measures.

## Author Contributions

SM, GJ, and VV conceived the research questions. VV carried out the experiments. SM, TH, HJ, and GJ supervised the project. VV, TH, SM, and HJ undertook the statistical analysis. All authors discussed and contributed to the interpretation of the results of the research and writing of the paper. All authors contributed equally to this work. All authors designed the experiments and research protocol.

### Conflict of Interest Statement

The authors declare that the research was conducted in the absence of any commercial or financial relationships that could be construed as a potential conflict of interest.
